# Prediction of True Circulatory Decompensation in Chronic Heart Failure for Optimal Timing of Mechanical Circulatory Support: Non-Invasive Arterial-Ventricular Coupling

**DOI:** 10.3390/jfb3010100

**Published:** 2012-02-01

**Authors:** Henryk Siniawski, Hans Lehmkuhl, Michael Dandel, Axel Unbehaun, Dagmar Kemper, Yuguo Weng, Roland Hetzer

**Affiliations:** Department of Cardiothoracic and Vascular Surgery, Deutsches Herzzentrum, Augustenburger Platz 1, Berlin 13353, Germany

**Keywords:** wave intensity, end-stage cardiomyopathy, heart failure

## Abstract

Background: Prospective comparative studies to predict the risk of hemodynamic deterioration in patients referred for transplantation were performed on the basis of standard invasive and non-invasive data and new wave intensity (WI) parameters. Methods and results: Study Group 1 consisted of 151 consecutive outpatients (age 48.7 ± 12 years; 110 men) with end-stage dilative cardiomyopathy. Group 2, consisting of 11 consecutive patients (age 50 ± 11 years; 6 men) with sinus rhythm and “true” decompensation, was used to create “critical values” of WI. There were no demographic or somatic (weight and height) differences between the groups. The follow-up period of ambulatory patients was 31 ± 8 months. Non-invasive WI was studied in the common carotid artery. Complete invasive and non-invasive data were also recorded on the day of investigation. During follow-up 44 pts were lost; there were 15 cardiac deaths (10%), life-saving ventricular assist device implantation in 10 (6.6%) and transplantation in 19 (12.7%). For statistical purposes this group was named the “events” Group B (*n* = 44). A predisposing factor for events (death, “true” decompensation and “urgent” transplantation in ambulatory patients) was low first peak (“cut-off value” assessed in Group 2 < 4100 mmHg*s³) (OR 45.6, CI 14.5–143.3, *p* < 0.001). Less powerful predictors of the risk of deterioration were pulmonary capillary pressure (PCP), diastolic pulmonary artery pressure (PAP) and E/A mitral wave relation (*p* = 0.05). Conclusions: The new ventricular-arterial coupling parameter 1st peak of WI can potentially be used to distinguish patients at high risk for true deterioration and death. This parameter can be used to predict the need for assist device implantation.

## 1. Background

Despite the large number of published papers dealing with the pathology of heart failure and great efforts to produce clinical guidelines [1,2], the question of how to select patients for adequate treatment (transplantation or other means) is not practically solved. There are no optimal clinical markers to monitor heart failure patients who constantly remain at risk of sudden und very often unpredictable deterioration. The cardio-respiratory symptoms [3], when they develop, are ominous signs occurring shortly before “true decompensation” so that the clinical picture is not very helpful for following this particular group of patients. Also, the systolic function of the left ventricle is no longer of value in predicting survival of the patients [4,5]. The function of the right heart chamber and pulmonary pressure, however, are better factors that predict mortality, but their practical value is limited [6,7]. 

Newly published data obtained in patients suffering from congestive heart failure (CHF) with preserved systolic function indicate that the cause of diastolic dysfunction possibly lies in arterial stiffness rather than in heart muscle dysfunction [8]. 

The interaction between the heart as a pump and the arterial conduit in congestive heart failure is seldom presented in the literature. The relation between the effect of the left chamber work, defined as stroke, and the arterial tree has been published by us as the results of an initial study with very promising results [9].

We tested the hypothesis that in patients with severely reduced systolic function “arterial incompetence” or poor mechano-hydraulic cooperation between the arterial conduit and the heart as a pump is an important factor and a sign that can precisely predict true decompensation (decompensation that cannot be treated by medical means only). 

This study presents our experience with wave intensity (WI) as an important parameter [10] for prognosis in patients suffering from end-stage dilative cardiomyopathy (DCM).

## 2. Patients and Methods

### 2.1. Patients

**Group 1** consisted of 151 consecutive outpatients (age 48.7 ± 12 years; 110 men) with end-stage DCM who fulfilled the inclusion criteria and were studied between 07/2001 and 12/2008 out of a mean of 182 patients/year referred for transplantation to the Deutsches Herzzentrum Berlin. Treatment included angiotensin-converting enzyme, diuretics, digoxin and beta-blockers in accordance with our institutional strategy. The results of the study did not influence the mode of treatment. Inclusion criteria were as follows: sinus rhythm, normal body mass index, no calcification or stenosis in the carotid artery studied, and mitral regurgitation of less than grade 1. The follow-up period was 31 ± 8 months.

**Group 2** consisted of 11 consecutive patients (age 50 ± 11 years; 6 men) with sinus rhythm and “true decompensation”, admitted for assist device implantation. This group is characterized by frank pulmonary decompensation and is qualified as Group “D” according to the ACC/AHA guidelines. The patients were studied before large doses of catecholamines were used to stabilize their hemodynamic condition. However, during the course of treatment decompensation appeared to be refractory to catecholamines and no medical means were able to keep the patients alive. All had to be treated by implantation of a ventricular assist device (VAD) to save their lives. The baseline characteristics of the patients are shown in Table 1. There were no demographic or somatic (weight and height) differences between the groups. Sinus rhythm was an inclusion criterion for the study. A maximal oxygen consumption study was performed during the same investigations only in Group 1.

**Table 1:** Baseline characteristics of the study groups.

	Characteristics	Group 1 (ambulatory VAD patients) *N* = 151	Group 2 (patients with true decompensation) *N* = 11	p
1	Age, *yrs*	47 ± 11	50 ± 11	0.48
2	Height, cm	175 ± 8	178 ± 11	0.2
3	Weight, kg	79 ± 15	75 ± 10	0.46
4	Dilative cardiomyopathy, *n*	150	11	
5	Duration of heart failure symptoms (years)	5.7 ± 3.3	5.9 ± 3.4	0.32
6	Number of decompensations	1.5 ± 0.06	1.6 ± 0.9	0,4
7	Serum sodium, mmol/L	136 ± 6.1	134 ± 5.8	0.2
8	GOT, u/L	15.1 ± 7.1	17.7 ± 8.2	0.2
9	LDH, u/L	76 ± 42	209 ± 42	0.001
10	Serum troponin, n/L	negative	0.54 ± 0.11	-
	**Hemodynamic data**			
11	PAP mean, mmHg	27 ± 11	36 ± 6	0.003
12	PCP, mmHg	18 ± 10	27 ± 3.3	0.0002
13	CI mL/min/m²	2.4 ± 0.81	2.0 ± 0.3	0.09

GOT, glutamate-oxalacetate transaminase; LDH, lactate dehydrogenase; PAP, pulmonary artery pressure; PCP, pulmonary capillary pressure; CI, cardiac index.

### 2.2. Methods

**Figure 1:**
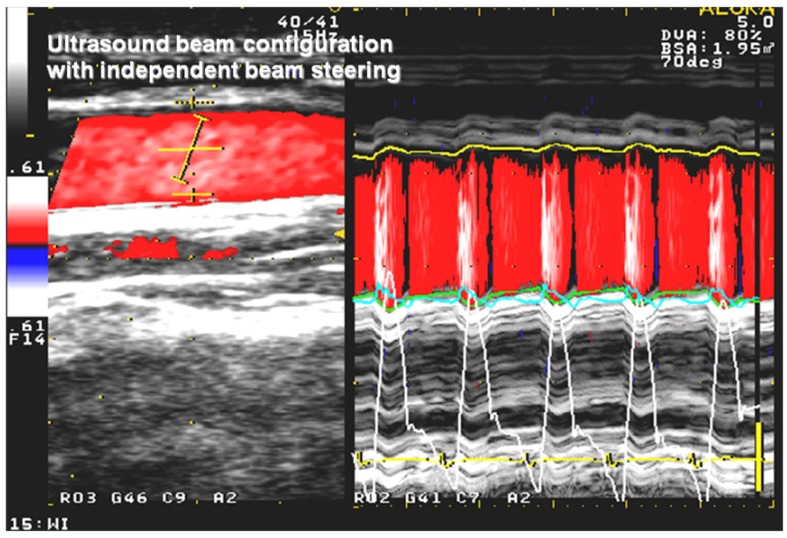
Echocardiogram of carotid artery with color flow and echo tracking beam localization (left side) and the diameter change waveform as the difference between posterior and anterior wall displacement (right side) assessed on the basis of echo tracking of the carotid artery calculated in real time (white curve for anterior wall, green for posterior wall) The white pressure-like configured curve corresponds closely to momentum of blood pressure.

The invasive wave intensity (WI) index is well described by Parker *et al*. [11] and was primarily calculated from invasive parameters as the product of the derivatives of velocity inside the artery and the pressure of the same artery. Non-invasive wave intensity (NWI) is defined on the basis of echocardiography (ALOKA ultrasonic system, Tokyo, Japan) and uses the protocol proposed by Sugawara *et al*. [12] and Harada *et al*. [10]. The pressure waveform is derived from the diameter change curve of the arterial wall. Assessment of the diameter curve is based on echo tracking beam configuration technology with independent beam steering of the carotid artery. Waveform (dU) is calculated in real time as the difference between posterior and anterior wall displacement during short time intervals (5 ms) (Figure 1) (ALOKA, Tokyo, Japan). The waveform (dU) closely corresponds to pressure change (dP) in a carotid artery measured invasively with high correlation quotient (goodness of fit r² = 0.97) and can be substituted for the invasively recorded pressure waveform [12]. Velocity measurements were based on 5 MHz continuous wave Doppler integrated equipment inside the studied artery at the same time as diameter changes. The formula for NWI is as follows:

NWI = (dU/dt) • (dV Doppler/dt)

dU—difference in displacement of carotid artery; V—flow velocity measured by Doppler.

**Figure 2:**
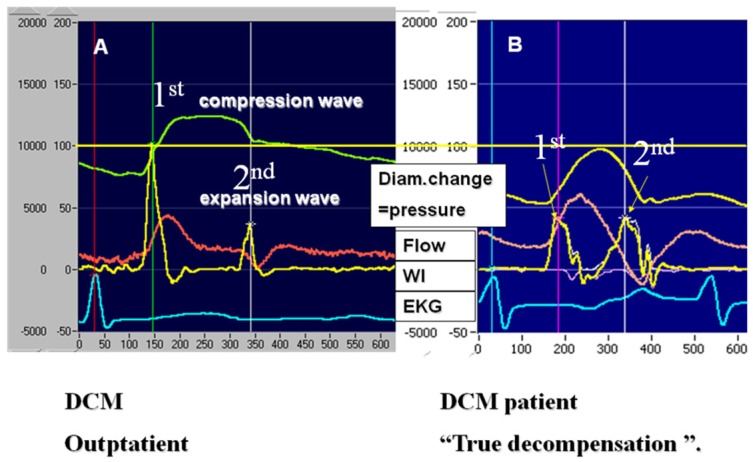
A and B. The non-invasive wave intensity (WI) index presented as the yellow curve consists of two peaks (1st and 2nd). The 1st peak (“compression wave”) is positive as a product of two positive values of pressure (green curve) and flow waveform (red curve). The height of the 1st peak depends on the value of pressure and flow and their dynamics at the beginning of ejection. The 2nd peak of WI (“expansion wave”), calculated like 1st peak, is the product of pressure and flow inside the vessel after ejection and represents reactivity of the vessel conduit in later phase. A is from a stable ambulatory patient and B from a patient during decompensation; the scale is the same for both patients.

The NWI index materializes as the curve consisting of two mathematically defined peaks (1st and 2nd). The 1st peak is the “compression wave” and is mathematically positive as a product of two positive values of pressure (green curve, Figure 2) and flow waveform (red curve). The compression wave describes the volume of the blood ejected in systole from the left ventricle conduit (aorta and arterial vessels). The 2nd peak of WI (“expansion wave”) is calculated in the same way as the 1st peak, which means that it is the result of algebraically calculated pressure and flow inside the vessel after ejection. While both curves (pressure and flow) in this phase are negative, algebraically their derivates produce positive peak of WI. The WI curve is recorded in yellow. The height of the 1st peak depends on the value of pressure and flow and their dynamics at the beginning of ejection (Figure 2).

### 2.3. Echocardiography

All patients received serial echocardiography, and were followed up according to our institutional protocol published elsewhere [13]. In patients who were hemodynamically instable echocardiography was performed daily (Group 2). 

*Pulsed Doppler mitral flow analysis*. In each patient, LV diastole flow velocity waves from five cardiac cycles were recorded and averaged. The following measurements were obtained: peak velocity of early diastolic filling wave (E); peak velocity of late filling (A). 

### 2.4. Proposed Heart Failure Values for 1st and 2nd Peak

Values of 1st and 2nd peak calculated in the truly decompensated group with added standard deviation (SD) were used as reference values to identify the risk of true decompensation (cut-off for 1st peak value at 4,100 mmHg*s³). For the purposes of this analysis these values were accepted for identification of patients at risk for “events” (death, true decompensation requiring assist device support or urgent transplantation).

### 2.5. Statistical Analysis

Demographic data and other characteristics are shown as number, as mean or as median ± SD.

The hypothesis was that the hemodynamic deterioration leads to three clinical situations, death, assist device implantation or urgent heart transplantation, and these situations were primary endpoints (events for statistical purposes). 

The second hypothesis was set that the value of 1st and 2nd WI peak assessed in the group of critically ill patients (*n* = 11) during true decompensation could be used as a “value of risk for true decompensation” in the ambulatory patients and would statistically correspond with the clinical endpoints of ambulatory patients. 

A cumulative survival curve was created to express the results of follow-up. Statistical significance was assumed for *P* values < 0.05. 

## 3. Results

### 3.1. Clinical, Invasive and Non-Invasive Characteristics

The baseline clinical characteristics of the two groups of patients studied are outlined in Table 1. The patients do not vary in terms of the duration of congestive heart failure symptoms or number of decompensations. The height and weight did not differ statistically between the groups.

The mean left ventricular filling pressure and mean pulmonary artery pressure (PAP) in Group 2 (during decompensation) were significantly lower compared to those of Group 1 and were 18 ± 9.81 *vs.* 27 ± 11 (*p* < 0.001) and 20 ± 10 *vs.* 28.6 ± 6 mmHg (*p* < 0.001), respectively. Cardiac index was higher in Group 1 and was 2.4 ± 0.8 L/min/m² *vs.* 2.0 ± 0.3 (*p* < 0.05), respectively. There was a difference between the groups regarding the serum level of enzymes: whereas Group 1 had normal levels of enzymes, the serum troponin was slightly elevated in Group 2 (0.54 ± 0.11 IU) without any other signs of infarction, as was LDH (209 ± 42 IU) and this enzyme elevation was probably the reason that hemodynamic decompensation was present.

The stable ambulatory group of patients (Group 1) compared to Group 2 did not have significantly smaller median LV diameter: 72 (min. 61, max. 100) mm *vs.* 75 (min. 60, max. 90) mm and median EF was also similar: 20 (min. 10, max. 40)% *vs.* 19 (min. 15, max. 25)% (Table 2). 

**Table 2:** Non-invasive data: LV dimension, ejection fraction, WI 1st, 2nd and β value, mean instantaneous maximal and minimal pressure and velocity in right carotid artery of stable ambulatory patients (Group 1, *n* = 150) and truly decompensated patients (Group 2, *n* = 11).

	*Echocardiography* median (min-max)	Group 1 (ambulatory VAD patients) *N* = 151	Group 2 (patients with true decompensation) *N* = 11	p
1	Heart rate, beats/min	77 (50–130)	89 (69–110)	0.01
2	LVEDD, mm	72.0 (51–100)	75.0 (60–90)	0.43
3	LVEF, %	20.0 (10–40)	19.0 (15–25)	0.1
	Non-invasive instantaneous mean values (carotid artery)*			
4	Pressure max. median (min-max), mmHg	104 (76–151)	84 (75–114)	0.007
5	Pressure min. median (min-max), mmHg	64 (36–100)	60 (38–72)	0.44
6	Velocity max. median (min-max), m/s	0.60 (0.22–1.47)	0.47 (0.91–0.47)	0.03
7	Velocity min. median (min-max), m/s	0.01 (−0.03–0.24)	0.007 (−0.8–0.09)	0.3
8	1st WI peak, mean ± SD	5.400 ± 3.500	2.900 ± 1200	<0.001
9	2nd WI peak, mean ± SD	1900 ± 1200	1800 ± 900	0.25
10	Β value, mean ± SD	15 ± 7	15 ± 7	0.2

* Values assessed during WI investigation; WI = wave intensity 1st and 2nd peak (mmHg m/s^3^); LVEDD, left ventricular end-diastolic diameter; LVEF, left ventricular ejection fraction

The differences between groups mentioned above were to be expected by the design of the study. The data were obtained from patients during frank decompensation (Group 2) where the hemodynamics per se was characterized by high filling pressures. The LV dimensions and systolic function (EF) did not differ significantly between Groups 1 and 2 and were: 72.0 (51–100) *vs.* 75.0 (60–90) mm, *p* = 0.43, and 20.0 (10–40) *vs.* 1 .0 (15–25)%, p = 0.1, respectively.

Non-invasive WI data obtained from the carotid artery including the values of 1st and 2nd WI peak and ß value are listed in Table 2. The truly decompensated group (Group 2) had significantly lower 1st peak than the stable patients (Group 1): 2.900 ± 900 *vs.* 5400 ± 3.500 mmHg*s³ (p < 0.001), while the values of the 2nd peak and ß values did not differ significantly between the groups and were for 2nd peak 1800 ± 900 and 1.900 ± 1200 and for ß value 15 ± 7 for both groups.

### 3.2. Follow-Up of Ambulatory Patients

Patients in Group 1 (*n* = 151) during a mean follow-up period of 31 ± 8 months experienced the following events which were defined as end-points for statistical purposes: 15 cardiac deaths (9.9%) before a VAD could be implanted to save the patients’ lives and 10 (6.6%) VAD implantations (true decompensation where stabilization by clinical means was not possible); 19 (12.6%) other patients received heart transplantation because of clinical deterioration. On the basis of these findings the patients were divided into two groups: event-free, Group A (*n* = 107) (stable ambulatory patients during follow-up) and event-positive, Group B (*n* = 44). These groups of patients were extensively studied to identify prognostic risk factors (Table 3). 

**Table 3:** Invasive and non-invasive characteristics of 150 studied patients. Group A event free and Group B event positive subgroup of patients (event = death, assist device implantation, transplantation).

		Group A *N* = 107 median (min-max)	Group B *N* = 44 median (min-max)	p
	***Invasive Data***			
	Heart rate, beats/min	77 (53–127)	75 (54–129)	0.42
1	PAP mean, mmHg	26 (8–55)	25 (13–51)	0.41
2	PAP diast., mmHg	18 (14–45)	21 (13–53)	0.069
3	CI, mL/min/m²	2.4 (1.3–5.2)	2.2 (1.1–3.6)	0.13
4	SV, mL	47.1 (19–108)	39.5 (10–71)	0.042
5	PCP, mmHg	17.0 (4–36)	19.0 (7–40)	0.058
	***Echocardiography***			
1	LVEDD, mm	72.0 (51–100)	77.0 (55–95)	0.27
2	LVEF, %	20.0 (10–40)	20.0 (10–35)	0.017
3	Mitral flow E/A	1.24 (0.01–2.9)	1.8 (0.1–9.0)	0.05
	***Exercise test***			
1	VO2 max., mL/min	17.6 (9–35)	13.9 (4–31)	0.037
	Non-invasive instantaneous mean values ( carotid artery)*			
1	Heart rate beats/min	73.9 (64–118)	77.2 (68–130)	0.65
2	BP max., mmHg	106 (76–145)	104 (71–141)	0.23
3	BP min., mmHg	64 (36–100)	63.5 (42–86)	0.3
4	Velocity max., m/s	0.626 (0.230–1.469)	0.524 (0.216–1.1215)	0.057
5	Velocity min., m/s	0.013 (−0.303–0.235)	0.023 (−0.85–0.153)	0.2
6	1st peak WI mean ± SD	6400 ± 3.300	2.900 ± 1200	<0.001
7	2nd peak WI mean ± SD	2000 ± 900	1400 ± 800	0.15

* Values assessed during WI investigation. PAP, pulmonary artery pressure; CI, cardiac index; SV, stroke volume; PCP, pulmonary capillary pressure; LVEDD, left ventricular end-diastolic diameter; LVEF, left ventricular ejection fraction; BP, blood pressure.

Invasively assessed systolic pulmonary artery pressure (PAP) did not vary significantly between Groups A (event-free) and B (event-positive); however the diastolic PAP and left filling pressure were elevated in the event-positive group: median 18 (14–45) *vs.* 21 (13–53) mmHg and 17.0 (4–36) (*p* = 0.069) *vs.* 19.0 (7–40) mmHg (*p* = 0.058), respectively. The difference in stroke volume (SV) between the two groups did not reach statistical significance; the median was 47.1 (19–108) mL *vs.* 39.5 (7–40) mL (*p* = 0.042), respectively.

There were no significant differences between the event-free and event-positive groups regarding echocardiographic parameters (EDD, EF and mitral flow pattern). Median EF was identical in the two groups (20%).

**Figure 3:**
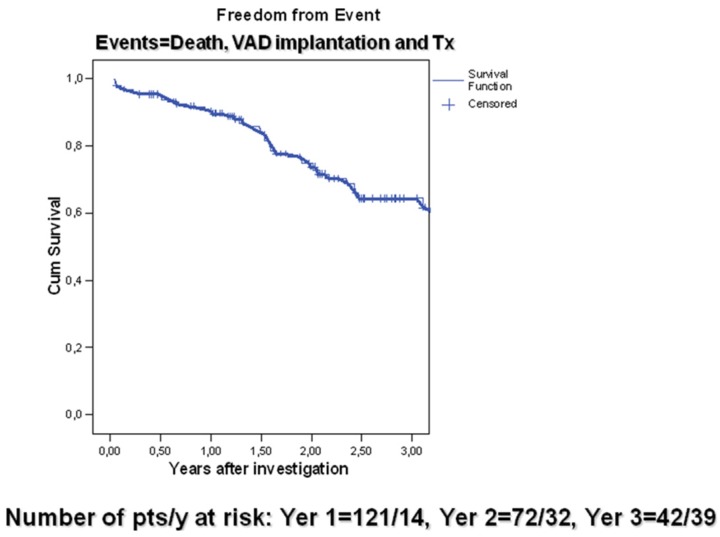
Event-free cumulative survival of ambulatory patients (*n* = 151). This curve does not represent a pure survival curve because patients who were removed from this curve were not only those who died but also those who were saved by transplantation or VAD implantation. Patients accepted in this way and regarded as “event free” represent those who were hemodynamically stable. (Death, VAD implantation and HTx (*n* = 44) in patients with DCM *n* = 150).

The 44 patients classified as having “events” (29.5%) and forming Group B were removed from follow-up to assess freedom from event and construct the survival curve (Figure 3). 

### 3.3. Wave Intensity Study

The mean values assessed in the carotid artery for NWI calculation were as follows:

heart rate and maximal pressures (systolic and diastolic) as well as diastolic minimal flow did not differ significantly between Group A and B; however the median value of maximal velocity in the carotid artery was significantly higher (*p* = 0.057) in the event-free patients (Group A) than in the “event” group (B) and was respectively 0.626 (0.230–1.469) and 0.524 m/s (0.216–1.1215) (Table 3).

**Figure 4:**
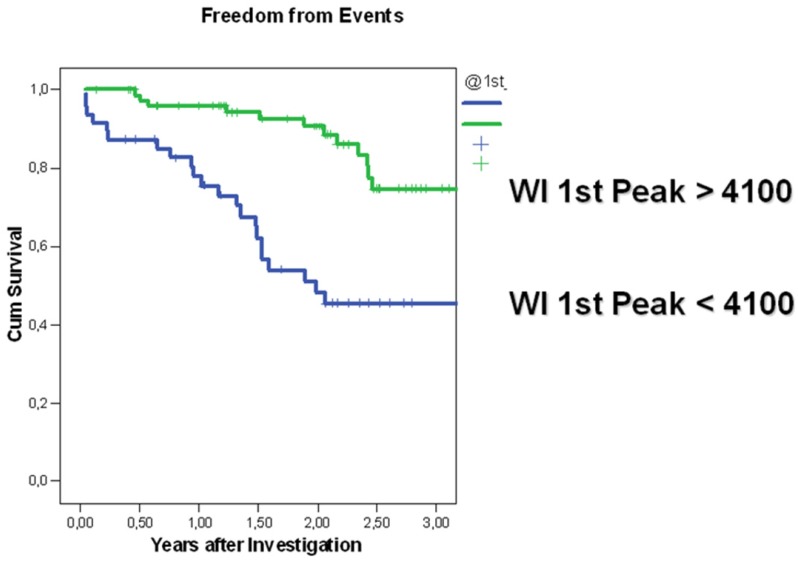
“Event free” cumulative survival for ambulatory patients (*n* = 151) divided into two groups according to the development of 1st peak WI: Survival of patients possessing WI 1st peak >4,100 mmHg*s³ (green curve); Survival with the peak <4,100 mmHg*s³ (blue curve). The cut-off value of 4,100 mmHg*s³ was established empirically by studying the group of patients during “true decompensation”. (Comparison of DCM groups of WI 1st peak).

Group A was characterized by 1st peak WI which was higher (6.400 ± 3.300) than the “value of risk for true decompensation” (<4,100 mmHg*s³) assessed by studying Group 1 during true decompensation state and Group B was characterized by 1st peak WI above such risk (>4,100 mmHg*s³). Patients in whom the assessed 1st peak was small (<4,100 mmHg*s³) had a significantly reduced chance of event-free survival compared with patients with 1st peak of 4.100 mmHg*s³ or more (OR 45.6, CI 14.5–143.3, *p* < 0.001). The 2nd peak did not differ significantly between the groups (Figure 4). 

## 4. Discussion

The usual and until now most plausible explanation for unexpected or unpredicted “true decompensation” was based on primary systolic or diastolic myocardial failure [14,15]. Little factual information is available regarding the function of the arterial system in the presence of “failing heart”; however the concept of a causal relation between heart and arterial conduit failure [16] seems to be an important topic. 

### 4.1. Measurement of WI Values for Decompensation Evaluation

The group of 11 consecutive patients (age 50 ± 11 years; 6 men) possessing sinus rhythm and being “truly decompensated” during chronic disease (duration of congestive symptoms was 5.9 ± 0.9 years with a mean of 1.6 ± 0.9 episodes of frank decompensation) were admitted for assist device implantation. We investigated the patients in hemodynamic cardiovascular shock but before norepinephrine was used; it had not been possible to stabilize them by medical means. This group was chosen as candidates for establishing “critical values” of peak 1st and 2nd and ß value of WI. 

### 4.2. Wave Intensity Parameters as Predictors of Hemodynamic Deterioration

Although NWI was assessed in the carotid artery it defines cooperation of the heart with the arterial conduit (aorta and arterial system) as a whole and not exclusively the arterial system of the head. This is the law of “connected compartments” (vessels). It seems to be true that for patients in whom heart dysfunction is present extracardiac factors may play an important role, leading to progressive decompensation; elevated filling pressure may cause heart dysfunction but may not always be the result of the loss of myocardial performance [17]. Newly published data supports the hypothesis that “hypervolemic episodes” in patients suffering from chronic heart failure could be the cause of decompensation events [18] rather than primary myocardial failure. This supports the results of our study that cooperation of the failing heart and arterial conduit can be an important factor for outcome prediction in patients suffering from congestive heart failure. Indeed, our prospective study demonstrates that 1st peak of WI which defines the systolic interaction of the arterial and ventricle system was highly significant for survival prediction if the 2nd peak was preserved. Preserved 2nd and low 1st peak has to be explained as a result of poor systolic function of the left chamber (small stroke volume and poor ejection dynamics) and poor response of the arterial system to ejected blood. Some research indicates that diastolic function of the heart may be reflected in the 2nd peak [19]; this could also be interpreted as high resistance of the vessel conduit causing diastolic dysfunction. It is believed that interaction or coupling is dependent on the “vigor” of the volume ejection into the aorta, producing the pressure wave equilibrium phenomenon forwards and backwards as well as the flow forwards and backwards inside the artery. Arterial stiffness factor ß was found to be also a good predictor of mortality in our group of patients but was not as prominent as 1st peak. High ß values (above 12) indicated patients at risk of death. There is no clear explanation of this phenomenon. However there were no differences in ß value between stable ambulatory patients and the truly decompensated patients (Table 1).

It should be kept in mind that the arterial tree functions as a “conductor” and plays a vital role as an active transmitter of blood supply to the peripheral tissue. Whether failure of this interaction has been provoked primarily by the steady failing of severely diseased myocardium or primarily by failure of the arterial tree could not be clarified; this was not the aim of our study. This study not only shows NWI to be a new clinical marker that is important for follow-up but also opens up new possibilities of using this new instrument to influence the medical treatment and the selection of specific treatment options. The reactivity of the arterial system to any kind of physiological stimulation is well known from animal studies [20]. However, patients suffering from congestive heart failure in general and specially during the advanced stages of the disease are very stable and do not demonstrate variability of WI parameters from beat to beat. This method was validated and demonstrated good reproducibility in human beings [21].

## 5. Critical Remarks and Conclusion

The ideal methods for assessment of contraction and relaxation properties during cardiac cycle would be the study of wave analysis on the basis of the LV chamber. Such studies have been performed in animal models and are based on invasive and non-invasive methods [22,23,24]. The study of the carotid artery has been considered as a surrogate for LV study by several researchers, as documented above. However, the critical point is how closely flow in the artery reflects flow in the aorta or LV chamber. Beyond this problem our work documented that non-invasive WI was a better prognostic marker of the risk of decompensation than traditional invasive and non-invasive factors.

Non-invasively assessed 1st peak of wave intensity (NWI) is a new, powerful marker with the strongest prediction (*p* < 0.001) of the risk of deterioration in patients suffering from chronic congestive cardiomyopathy. NWI is cost effective and uncomplicated in assessing the quality and quantity of cooperation between the heart and arterial system. This could help to separate the DCM patients who will remain stable in the long term from those with a high risk of progressive and so far unpredictable decompensation and death.

The invasive parameters (pulmonary capillary and diastolic pulmonary artery pressure) as well as relation of mitral filling waves (E/A) had less power of prediction (*p* = 0.05).
